# Characterization of chromosome 5 aberrations in *TP53* mutated myeloid neoplasms with ≥5% blasts: An International *TP53* Investigators Network (iTiN) study

**DOI:** 10.1002/cncr.70210

**Published:** 2025-12-19

**Authors:** Irfan Yasin, Anna Stengel, Haipeng Shao, Amandeep Kaur, Emily F. Mason, Pranav P. Patwardhan, Nathanael G. Bailey, Sharmila Ghosh, Kedar V. Inamdar, Anand A. Patel, Madhavi Pandiri, Jingjing Zhang, Payal Sojitra, Hamza Tariq, Zenggang Pan, Danica Wiredja, Peng Wang, Melissa Y. Tjota, Jeremy P. Segal, Hong Chang, David A. Sallman, Daniel A. Arber, Ayalew Tefferi, Talha Badar, Anamarija M. Perry, Claudia Haferlach, Angela M. Lager, Girish Venkataraman

**Affiliations:** ^1^ Pathology (Hematology/Oncology & Hematopathology & Genomic Pathology & Cytogenetics), Medicine (Hematology/Oncology) The University of Chicago Medicine Chicago Illinois USA; ^2^ Munich Leukemia Laboratory Munich Germany; ^3^ Pathology (Hematopathology), Medicine (Hematology Oncology) H. Lee Moffitt Cancer Center and Research Institute Tampa Florida USA; ^4^ Pathology (Molecular Pathology) Endeavor Health Northshore Hospital Evanston Illinois USA; ^5^ Pathology, Microbiology, and Immunology (Hematopathology) Vanderbilt University Medical Center Nashville Tennessee USA; ^6^ Pathology (Hematopathology) University of Pittsburgh Medical Center Pittsburgh Pennsylvania USA; ^7^ Pathology (Hematopathology) Mayo Clinic Rochester Minnesota USA; ^8^ Pathology (Hematopathology) Henry Ford Health System Detroit Michigan USA; ^9^ Pathology (Hematopathology & Children's Hospital of Colorado) UCHealth University of Colorado Hospital Anschutz Medical Campus Aurora Colorado USA; ^10^ Pathology Rutgers Robert Wood Johnson Medical School New Brunswick New Jersey USA; ^11^ Pathology (Hematopathology) Northwestern Memorial Hospital Chicago Illinois USA; ^12^ Laboratory Medicine Program (Laboratory Hematology) University of Toronto Toronto Ontario Canada; ^13^ Medicine (Division of Hematology) Mayo Clinic Rochester Minnesota USA; ^14^ Medicine (Hematology‐ Oncology) Mayo Clinic Jacksonville Florida USA; ^15^ Pathology (Hematopathology) University of Michigan Ann Arbor Minnesota USA

**Keywords:** *TP53*, MDS, AML, cytogenetics, pathology, outcomes

## Abstract

**Background:**

Isolated chromosome 5/5q losses (–5/5q) without *TP53* mutations are associated with favorable outcomes in myeloid neoplasms (MN) with <5% blasts. However, the clinical implication of concurrent −5/5q and *TP53* aberrations in MN with ≥5% blasts is poorly understood.

**Methods:**

Patients with *TP53*‐mutated MN carrying ≥5% blasts assessing the prognostic impact of ‐5/5q on 24‐month overall survival (OS24) were examined.

**Results:**

Of 587 patients, 515 (88%) exhibited −5/5q overwhelmingly in the context of a complex karyotype (98.3% vs. 61.1% complex karyotype without −5/5q; *p <* .0001) and multihit *TP53* allelic state (88.3% vs. 56.9%; *p <* .0001). Proportions of patients with blasts ≥20% were comparable between groups with and without −5/5q; *p =* 0.26. Notably, patients with −5/5q exhibited significantly fewer coalterations; *p <* .0001. Looking at outcomes, presence of −5/5q was associated with shorter median 24‐month overall survival (7.8 months vs. 11.2 months; *p*_*Log‐rank*_ = .012), an effect restricted to subgroups with blasts <20% (*p =* .039; *N =* 163), absent −7/7q (*p =* .007; *N =* 225), or WHO5‐defined single hit allelic state (*p =* 0.030; *N =* 91). Importantly, −5/5q retained independent adverse prognostic significance regardless of *TP53* allelic state in a multivariable model. Furthermore, among the subset of 75 (13%) patients undergoing allogeneic stem cell transplantation, −5/5q predicted significantly shorter median 5‐year posttransplant survival (16.2 months vs. median not reached; *p*_*Log‐rank*_ = .009).

**Conclusions:**

These findings emphasize the independent prognostic relevance of chromosome 5/5q losses underscoring the clinical relevance of cytogenetic testing for −5/5q even in this high‐risk cohort.

## Abstract 2

Chromosome 5 alterations in *TP53* mutated myeloid neoplasms are associated with complex karyotype and multi‐hit *TP53* allelic state and confer negative prognostic impact in subgroups with blasts <20% and single‐hit *TP53* allelic state.

## INTRODUCTION

Myelodysplastic syndromes (MDS) with isolated del(5q) (iso‐5q) carries a favorable prognosis in the absence of a monosomy 7 or complex karyotype (CK).^1^ Earlier studies evaluated the impact of *TP53* mutation in this disease,^2^, ^3^, ^4^ with recent data indicating that *TP53* monoallelic patients with a *TP53* variant allele frequency (VAF) < 20% carry largely similar outcomes as those with wild‐type *TP53*.^5^ However, the acquisition of progressive karyotypic complexity and biallelic *TP53* alterations superimposed on a del(5q) result in uniformly poor survival, making del(5q) seem clinically irrelevant once blast counts exceed 5%.^1^, ^6^, ^7^ Although there is a wealth of published literature attesting to the adverse prognostic effect of −7/7q^8^, ^9^ and del(17p),^10^, ^11^, ^12^, ^13^, ^14^, ^15^, ^16^ the independent impact of del(5q) within *TP53*‐mutated myeloid neoplasms outside the context of MDS with iso‐5q is poorly understood.

One study from 2012 looking at a high‐risk acute myeloid leukemia (AML) cohort showed a 49% 2‐year event‐free survival for isolated del(5q) with absent del(17p), but did not assess *TP53* mutations.^16^ A subsequent study that evaluated *TP53* mutations and impact of the type 5q losses linked unbalanced 5q losses to frequent *TP53* mutations and clonal evolution in MDS but not in AML.^17^ A very recent clonal hierarchy work by this group suggests two mechanisms of clonal evolution in MDS with isolated −5/5q. The first via *TP53* mutation accrued on top of an ancestral iso‐5q, or alternatively an ancestral *TP53* mutated clone acquiring subsequent del(5q).^18^ These studies raise a broader question: Does −5/5q still matter once *TP53* is mutated, and if so, does its effect vary by how 5q is lost? The question has gained urgency especially considering the new *TP53*‐mutated myeloid‐neoplasm categories proposed by WHO‐5^1^ and the International Consensus Classification.^7^

With this background, we analyzed a cohort of MDS‐IB1,^1^ MDS‐EB,^7^ and AML with ≥ 1 pathogenic *TP53* mutation. We asked (1) whether del(5q) confers additional adverse risk across the blast spectrum and (2) whether its impact depends on interplay of *TP53* allelic state or karyotype. Among chromosome‐5‐loss cases, we also compared interstitial deletions (−5/5q^***i***^) with non‐interstitial lesions (−5/5q^***ni***^, encompassing unbalanced translocations, monosomies, and ring chromosomes) to determine whether the mechanism of 5q loss has prognostic relevance.

## MATERIALS AND METHODS

### Patient cohort

We included patients with a myeloid neoplasm with ≥5% blasts carrying one or more pathogenic/likely pathogenic *TP53* mutation at a VAF ≥3%. These included patients that would be classified as MDS‐EB or MDS with biallelic *TP53*, MDS/AML, and AML per the ICC schema diagnosed between 2009 and 2024.^7^ Patients were included regardless of the initial treatment approach excluding patients with a known MPN who acquired a *TP53* mutation at progression/acceleration. We collected detailed baseline pathology data, cytogenetic information (detailed later), somatic sequencing data, as well as clinical data including outcome.

### Cytogenetic analysis and assessment of −5/5q status

A combination of G‐banded chromosome analysis, fluorescence in situ hybridization (FISH), and next‐generation sequencing (NGS) was performed to assess for chromosome 5 copy number status. In 82% of cases, a full 20 metaphase cell analysis was performed. All karyotypes were standardized according to ISCN 2016.^19^ Parsing scripts were developed in Python 3.12 to extract the copy number state of chromosomes 5, 7, and 17 from the karyotype results. Interphase FISH analysis was performed with the EGR1 (5q31) and D5S23/D5S721 (5p15.2) DNA FISH probe set (Abbott Molecular), scoring 200 to 400 interphase nuclei per case. For 15% of cases, chromosome 5 copy number status was available via NGS. Chromosome 5 copy number status via NGS was used only when karyotype or FISH data were partially available and was not the sole source for assessing chromosome 5 copy number status. A total of 400 patients underwent testing at two consortium centers to ensure methodological consistency for cytogenetic methods. Cases with <3 G‐banded metaphases and missing FISH data were excluded.

### Somatic molecular testing

Somatic molecular testing was performed on diagnostic samples either on bone marrow or peripheral blood (within 1 month of the diagnostic bone marrow biopsy). Testing was performed either using hybrid‐capture or amplicon‐based sequencing method using panels specific to each institution as reported in our previous work.^20^ Copy number alteration data were also available for 20% of cases and reported alongside somatic alterations. Only shared genes across most centers are reported here and include most recurrently altered genes related to myeloid neoplasms. Although germline testing data were available in a small subset of patients, only gene alterations identified as being somatic in nature were modeled in this study.

### Statistical analysis

All statistical analyses were performed using Stata 18 (College Station, TX) or Python 3.12. Code for karyotype and somatic‐NGS string parsing/cleaning (Python 3.12, Stata 18) were iteratively adjusted with OpenAI ChatGPT (models 4o and o3). All artificial intelligence–generated code segments were manually reviewed and revised as necessary before use on full dataset. Continuous variables were summarized using median (range), whereas associations for binary frequency data were evaluated via chi‐square or Fisher exact tests, as appropriate. The primary survival endpoint was 24‐month overall survival (OS24), calculated from the date of diagnosis with censoring at 24 months. For the subgroup of patients receiving allogeneic stem cell transplantation, the survival endpoint extended from the transplant date to censoring at 60 months posttransplant. Kaplan–Meier product‐limit methods were employed to estimate survival distributions and differences assessed by log‐rank tests. Multivariable modeling used Cox proportional hazards regression or flexible parametric models^21^ (when the proportional hazards assumption was violated), with hazard ratios (HR) and 95% CIs reported. No imputation for missing values was conducted in any analysis.

## RESULTS

### Characteristics of the subgroup harboring −5/5q

**FIGURE 1:**
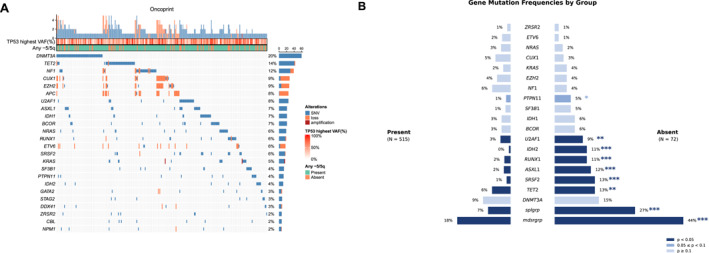
Co‐mutations and structural alterations in the cohort. (A) Oncoprint of the topmost frequent co‐alterations in entire cohort. *DNMT3A* and *TET2* alterations were predominant followed by copy number alterations of *NF1*, *CUX1*, *EZH2,* and *APC*. (B) Mirrored bar plot depicting frequencies of various co‐alterations in groups stratified by the 5/5q status. Overall, co‐occurring alterations were infrequent in the group with 5/5q, particularly the myelodysplasia‐related genes. Gene‐wise frequencies between subgroups were tested with two‐sided Fisher exact tests with depicted *p* values corrected for multiple comparisons using the Benjamini‐Hochberg procedure (false discovery rate, 5%).

Table 1 summarizes the baseline characteristics and treatment regimens of all patients, stratified by −5/5q status at *TP53* ^*MUT*^ myeloid neoplasm (MN) diagnosis. Individuals with −5/5q carried a lower median blast count of 27% versus 35.25 (*p =* .006) compared to those without −5/5q. Cytogenetic profiling showed that −5/5q was highly associated with complex karyotype (*p* < .0001) and frequently co‐occurred with −7/7q loss (*p* < .001), and multi‐hit *TP53* allelic state configuration (*TP53* ^*MH*^; *p* < .0001) including del(17p) (*p* < .0001), trisomy 8 (*p =* .041) as well as *TP53* VAF >50% (*p =* .004). Analysis of the entire cohort revealed *DNMT3A* and *TET2* mutations as the most frequent alterations (Figure 1A for oncoprint of top mutations in the cohort). Co‐occurring molecular alterations were infrequent in those with −5/5q compared to patients lacking −5/5q (48% vs. 76% without 5/5q; *p <* .0001) (see Figure 1B). In particular, the −5/5q cases lacked coalterations involving myelodysplasia‐related genes (18% vs. 44% with 5/5q; *p <* .0001) as well as spliceosome factor genes (28% vs. 8%; *p <* .0001)

**TABLE 1:** Baseline characteristics of all patients stratified by 5/5q status.

	No −5/5q	−5/5q	Test
	*N* = 72 (12.3%)	*N* = 515 (87.7%)	
Baseline laboratory values, median [IQR]			
Hemoglobin (g/dL)	8.8 [6–13]	8.1 [3–18]	0.001
Platelet count (10^3^/μL)	50.5 [6–271]	50.0 [1–585]	0.726
Abs. Neut. Count (10^3^/μL)	0.6 [0–32]	0.7 [0–21]	0.217
Age at diagnosis			
≤70 years	35 (48.6%)	238 (46.2%)	0.702
>70 years	37 (51.4%)	277 (53.8%)	
Gender			
Female	28 (38.9%)	236 (45.8%)	0.268
Male	44 (61.1%)	279 (54.2%)	
Blast count (WHO5) at diagnosis			
<20%	16 (22.2%)	147 (28.5%)	0.262
≥20%	56 (77.8%)	368 (71.5%)	
Therapy‐related vs. other (de novo, secondary)			
Not t‐MN	43 (64.2%)	333 (69.4%)	0.390
t‐MN	24 (35.8%)	147 (30.6%)	
Complex karyotype			
Absent	28 (38.9%)	9 (1.7%)	<0.001
Present	44 (61.1%)	506 (98.3%)	
Any −7/7q			
Absent	45 (62.5%)	180 (35.0%)	<0.001
Present	27 (37.5%)	335 (65.0%)	
Any del(17p)‐NGS‐FISH‐karyotype			
Absent	50 (69.4%)	209 (40.6%)	<0.001
Present	22 (30.6%)	306 (59.4%)	
No. of *TP53* mutations			
1 mutation	59 (81.9%)	383 (74.8%)	0.186
2+ mutations	13 (18.1%)	129 (25.2%)	
*TP53* VAF >25%			
≤25%	30 (41.7%)	78 (15.2%)	<0.001
>25%	42 (58.3%)	434 (84.8%)	
*TP53* allelic state^a^			
Single‐hit	31 (43.1%)	60 (11.7%)	<0.001
Multihit	41 (56.9%)	452 (88.3%)	
Co‐alteration. no. (%)^b^			
Absent	17 (23.6%)	267 (51.8%)	<0.001
Present	55 (76.4%)	248 (48.2%)	
MDS‐related gene mutation			
Absent	40 (55.6%)	420 (81.6%)	<0.001
Present	32 (44.4%)	95 (18.4%)	
EPI6 signature‐no. (%)^c^			
Absent EPI6	52 (72.2%)	426 (82.7%)	0.032
Present EPI6	20 (27.8%)	89 (17.3%)	
Therapies‐no. (%)			
Low‐intensity (HMA ± VEN)	49 (68.1%)	387 (77.1%)	0.010
Intensive (chemotherapy)	17 (23.6%)	51 (10.2%)	
Best supportive care	4 (5.6%)	42 (8.4%)	
CPX‐351/Vyxeos	2 (2.8%)	22 (4.4%)	
HMA therapy group‐no. (%)			
HMA without Ven^d^	23 (46.9%)	202 (53.2%)	0.412
HMA + Ven	26 (53.1%)	178 (46.8%)	

Abbreviations: FISH, fluorescence in situ hybridization; IQR, interquartile range; MDS, myelodysplastic syndromes; Med., median; NGS, next‐generation sequencing; t‐MN, therapy‐related myeloid neoplasms; VAF, variant allele frequency.

^a^ WHO5‐defined allelic state agnostic to *TP53* VAF.

^b^ Somatic alteration of genes aside from *TP53*.

^c^ EPI6 signature comprising a six‐gene co‐alteration signature (*EZH2*, *TET2*, *CUX1*, *CBL*, *NF1*, *KRAS*) described recently.

^d^ Besides HMA monotherapy, some patients in this group received combinations of decitabine/cedazuridine, or magrolimab.

### Interstitial versus non‐interstitial −5/5q

Among the patients with −5/5q (*N =* 515), −5/5q^***i***^ was predominant (478/515; 93%) whereas the −5/5q^***ni***^ group was significantly smaller (37/515; 7%). Compared to the −5/5q^***i***^ subgroup, a significantly smaller proportion with −5/5q^***ni***^ were older than 70 years (32% vs. 55%; *p =* .007). There were no differences in blast counts, *TP53* VAF, co‐alteration frequencies, or patterns between the two groups (41% vs. 49%; *p =* .34).

### Analysis of outcomes

#### Entire cohort analysis

**FIGURE 2:**
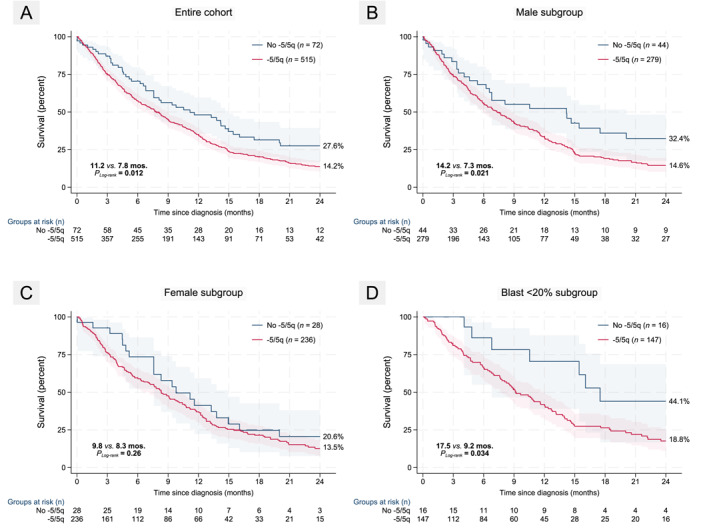
KaplanMeier plots depicting impact of −5/5q on 24‐month survival in entire cohort and various subgroups. (A) −5/5q predicts inferior outcome in entire cohort. (B, C) The adverse impact is restricted to male gender only. (D) When looking at blast count subgroups, impact is restricted to those with blasts 20%. Log‐rank *p* values are depicted besides median survival and 24‐month OS% to the right of each stratum in the graph.

**FIGURE 3:**
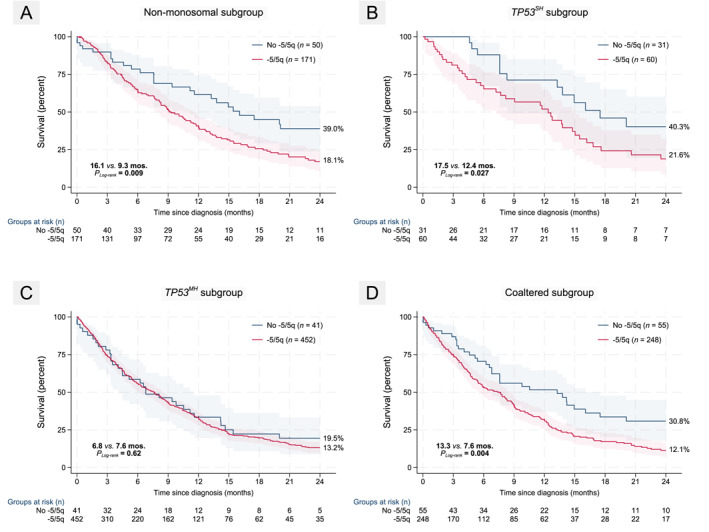
Kaplan–Meier plots depicting impact of −5/5q on 24‐month survival in additional key subgroups. (A) −5/5q was relevant only in those lacking a monosomal karyotype. (B, C) When examined within *TP53* allelic state subgroups, −5/5q remained relevant only in those with single‐hit allelic status but not in the multi‐hit allelic state subgroup indicating that other factors within a CK such as 7/7q and high *TP53* VAF are more relevant in determining outcome in clonally progressed cases. (D) Last, when examining only those patients with any somatic co‐alterations (i.e., aside from *TP53* mutations), −5/5q was again relevant for predicting inferior 24‐month survival. Log‐rank *p* values are depicted besides median survival and 24‐month OS% to the right of each stratum in the graph.

At a median follow‐up of 6 months (range, 0–91 months), 491 (78%) deaths and 79 (12%) alloSCTs were recorded with a 90‐day mortality of 23% (145/631 deaths); notably, the number of deaths was not higher among patients carrying −5/5q (79.0% vs. 70.8%; *p =* 0.12). In the OS24 analysis, −5/5q conferred inferior median OS24 (13.3% vs. 25.8%; *p*_*fpm*_ = .011) with impact restricted to those with blasts <20% (HR, 2.2 [1.0–4.8]; *p =* .039; *N =* 163) (see Figure 2). Given the adverse prognostic significance of a monosomal karyotype (using a modified definition including 0‐1 vs. 2+ autosomal monosomies) reported previously by our group in *TP53* ^*MUT*^ MNs, we examined impact of −5/5q separately in each subgroup. See Figure 3A for relevance of −5/5q in only those with 0 to 1 monosomies.

#### Impact of −5/5q is restricted to those with single‐hit *TP53* allelic state

Because −5/5q travels largely with a CK, which independently confers poor prognosis anyway, we asked if the impact is differential by WHO5‐defined allelic state. In the subgroup analysis of *TP53* ^*SH*^ individuals, 63 of 91 (69%) carried a CK, and −5/5q predicted adverse outcome (HR, 2.0 [1.1–3.8]; *p =* .030; *N =* 91) within the *TP53* ^*SH*^ subgroup as well. There was no impact in the subgroup within the *TP53* ^*MH*^ subgroup (HR, 1.1 [0.8–1.6]; *p =* .62; *N =* 493) (see Figure 3). Further stratification by del(17p) status showed that the adverse effect of −5/5q was confined to patients lacking del(17p) (HR, 1.7 [1.1–2.5]; *p =* .015; *N =* 259). As expected, −5/5q also impacted outcomes only in those with a single *TP53* mutation (HR, 1.7 [1.2–2.4]; *p =* .003; *N =* 442) but not in those with more than one *TP53* mutation (HR, 0.8 [0.4–1.5]; *p =* .43; *N =* 142). Given the relevance of −7/7q as an exclusion criterion in defining MDS and iso‐5q, we looked at impact of −5/5q within each stratum by −7/7q status. As expected, −5/5q predicted significantly inferior OS24 only in patients lacking a −7/7q (HR, 1.9 [1.2–2.9]; *p =* .007; *N =* 225) but not in those with harboring a concurrent −7/7q (HR, 1.0 [0.6–1.5]; *p =* .93; *N =* 362). These findings indicate that −5/5q has prognostic relevance only in patients with lower blast counts, single‐hit allelic state, or lacking −7/7q supporting the validity of WHO5‐ and ICC‐defined *TP53* allelic state classifications as well as the individual measures of *TP53* allelic state configuration.

#### Interstitial versus non‐interstitial losses of chromosome 5

We next asked if all forms of chromosome 5 alteration carry similar prognostic relevance. We compared the impact of −5/5q^***ni***^ versus −5/5q^***i***^ on OS24 and found no significant difference (HR, 1.0 [0.7–1.4]; *p =* .92). Because the relevance of −5/5q appears to be greater in less clonally evolved subgroups (non‐CK), we conducted separate analyses looking at interstitial and non‐interstitial losses within subgroups based on *TP53* allelic state configuration. Again, there was no difference in outcome between these two groups. Last, we looked at subgroups defined by *TP53* VAF% examining cutoffs of 25% and 50%. In the subgroup with VAF ≤25%, −5/5q^***ni***^ exhibited inferior outcomes compared to −5/5q^***i***^ in the subgroups VAF ≤25% (HR, 2.6 [1.1–5.9]; *p =* .022; *N =* 78). Structural chromosome 5 losses did not impact outcome in either of the high VAF subgroups including VAF >25% (*p =* .33) or VAF >50% (*p =* .81).

#### Impact in allotransplanted patients

Last, we analyzed the 79 (12%) patients who underwent alloSCT evaluating 60‐month posttransplant overall survival (OS60). There was no difference in OS60 between MDS (blasts <20%) and AML (16.4% vs. 15.5% in AML; *p*_*Log‐rank*_ = .26 [*N =* 79]). While alloSCT rates did not differ significantly by −5/5q status, −5/5q was associated with worse posttransplant outcomes (HR, 4.2 [1.3–13.6]; *p =* .016; *N =* 75). It is likely that this is merely a reflection of the overarching predominance of complex karyotype in our cohort. Surprisingly, *TP53* ^*MH*^ configuration (regardless of WHO5 or ICC definitions) did not impact survival in transplanted patients (16.6 months vs. 26.3 months; *p*_*Log‐rank*_ = .82). We next assessed impact of −5/5q within each allelic state subgroup. In this analysis, −5/5q predicted inferior median 5‐year posttransplant survival only in the *TP53* ^*MH*^ subgroup (16.2 months vs. median not reached; *p*_*Log‐rank*_ = .048). The analysis of the *TP53* ^*SH*^ transplanted subgroup (*N =* 14) was too small for a well‐powered analysis and hence not undertaken.

#### Transplant‐stratified multivariable model supports independent prognostic relevance of −5/5q

Given the overarching predominance of complex karyotype in the cohort, we asked if −5/5q has independent prognostic relevance in a multivariable model including pretherapy predictors, particularly in conjunction with allelic state determinants. We constructed a transplant‐stratified multivariable model including −5/5q with other candidate predictors, WHO‐defined *TP53* allelic state adjusting the model for age >70 years at diagnosis as well as gender. We specifically chose not to include CK or −7/7q in the model given their high correlation with −5/5q. Presence of −5/5q retained independent predictive ability for 24 months survival in both uni‐ and multivariable model besides the WHO‐defined allelic state of *TP53* supporting its independent prognostic ability (see Table 2).

**TABLE 2:** Stratified multivariable Cox proportional hazards regression of key pretherapy predictors of outcomes.

	Univariable Cox		Multivariable Cox	
	HR	95% CI	HR	95% CI
Age at diagnosis				
≤70 years	1.00		1.00	
>70 years	0.89	[0.74–1.09]	0.89	[0.76–1.04]
Gender				
Female	1.00		1.00	
Male	1.04	[0.88–1.22]	0.99	[0.85–1.16]
WHO5 *TP53* allelic state				
Single‐hit	1.00		1.00	
Multihit	1.45^b^	[1.21–1.75]	1.54^b^	[1.21–1.97]
Any −5/5q				
Absent	1.00		1.00	
Present	1.40^b^	[1.20–1.63]	1.19^a^	[1.01–1.40]

Data stratified by transplant status in uni‐ and multivariable Cox regression models including *TP53* allelic state and determinants besides −5/5q with standard errors adjusted for variance across centers. −5/5q remained significant in this stratified analysis in the uni‐ and multivariable models in addition to WHO5‐defined *TP53* allelic state. Hazard ratio (HR) and 95% CIs of HR using Cox Proportional Hazards regression with a clustered sandwich estimator for centers.

*p < .1.

^a^ *p* < .05.

^b^ *p* < .01.

## DISCUSSION

In this large multicenter cohort looking at *TP53* mutated myeloid neoplasm, we identified that −5/5q frequently co‐occurs in the background of a CK and *TP53* ^*MH*^ allelic state, aligning with prior evidence that del(5q) often marks clonal evolution in myeloid malignancies.^22^ The large size of the cohort enabled detailed subgroup analyses where the adverse prognostic effect of del(5q) was largely limited to patients lacking CK or del(17p), suggesting that a CK dominates outcome, diminishing any independent contribution of del(5q) in clonally progressed cases. Further subset analysis by blast count confirmed that the impact of any concurrent −5/5q may be relevant only in patients with <20% blasts and those with single‐hit allelic state.

In the analysis of allo‐transplanted patients, alloSCT significantly improved outcomes in both MDS as well as AML subgroups in line recent data from Senapati and coworkers^23^ in *TP53* mutated MDS and AML. Although we noted no difference in allogeneic transplantation rates by −5/5q status, we surprisingly found that −5/5q (but not −7/7q or –17/17p) adversely impacted posttransplant survival. This finding is congruent with an earlier study by Middeke and coworkers in a high‐risk AML cohort indicating favorable posttransplant 2‐year EFS with isolated del(5q) lacking del(17p),^16^ whereas a more recent EBMT study showed adverse impact of 5q in the context of an abnormal 17(p) or monosomal karyotype,^24^ although neither study evaluated *TP53* mutations. Overall, these studies and our collective data emphasize that the impact of del(5q) is indeed context dependent and carries prognostic value in transplanted individuals.

On the other hand, there has been interest in assessing the impact of the nature of losses on chromosome 5 (interstitial vs. non‐interstitial). A prior study of 1200 patients with MDS or AML evaluated the spectrum and impact of the types of chromosome 5 losses (interstitial deletions involving commonly deleted regions [CDR2/CDR1] vs. unbalanced translocations involving losses of the commonly retained regions in the paracentromeric region and telomeric region distal to band 5q34).^17^ This study noted that patients with MDS (but not AML) within those harboring non‐interstitial losses were more likely to harbor *TP53* mutations and adverse outcomes related to frequent clonal evolution. While we recapitulated this analysis looking at the impact of both types of structural losses, we did not see any impact in the overall cohort, although there was an adverse effect only within the subgroup with VAF ≤25%. These differences are likely explained by differences in cohort characteristics with exclusive *TP53* mutations and high frequency of complex karyotype in our cohort. Despite the cohort size, our analysis was admittedly underpowered this last analysis given the paucity of patients in the non‐interstitial‐only subgroup.

On the other hand, the biological mechanisms underlying the impact of del(5q) remain incompletely understood, though prior studies have implicated the loss of key genes within the CDR on 5q. Haploinsufficiency of genes such as RPS14 and EGR1, and microRNAs such as miR‐145/146a has been shown to impair erythroid differentiation and immune regulation, potentially contributing to disease progression.^25^, ^26^, ^27^ A potential mechanism involves the therapeutic use of lenalidomide, a mainstay in treating del(5q) MDS. Lenalidomide exerts its effect by promoting casein kinase 1A1 degradation and inducing apoptosis—an effect dependent on functional *TP53*. In *TP53*‐mutated cells, this apoptotic pathway is impaired, resulting in lenalidomide resistance. Consequently, lenalidomide may create a selective pressure that spares *TP53*‐mutant clones while suppressing others, facilitating clonal expansion, disease progression, and eventual transformation to therapy‐related myeloid neoplasms.^28^, ^29^ This interplay between genetic deletion and *TP53* dysfunction likely contributes to more aggressive disease phenotypes and inferior clinical outcomes.

While del(5q) in the absence of a complex karyotype is traditionally regarded as a favorable cytogenetic feature in MDS, as reflected in IPSS‐M,^30^, ^31^ our results challenge this paradigm in *TP53*‐mutated cases with ≥5% blasts. In this context, del(5q) is associated with inferior survival—though less severe than outcomes linked to −7/7q—highlighting the nuanced prognostic implications of −5/5q when considered alongside blast burden, karyotypic complexity, and *TP53* status. Although a CK with −7/7q likely overshadows the negative impact of −5/5q in *TP53*‐mutated cases, our findings nonetheless support the WHO5 and ICC rationale behind excluding concurrent −7/7q from the definition of MDS with isolated del(5q).^1^ That said, we believe that estimation of allelic state is not practically feasible in real time for clinical practice and that an allelic state measure agnostic to NGS‐based LOH may be more suitable for *TP53* mutated myeloid neoplasms more so only in the absence of a CK.

The key strength of this study lies in the well‐annotated karyotype and NGS data, which enabled sufficiently powered and detailed subset analyses. As a multicenter retrospective study, however, it is subject to inherent limitations, including potential selection bias, missing data, and variability in sequencing platforms, cytogenetic assessment, and treatment approaches across sites. The <5% blasts with complex karyotype subgroup lacked sufficient numbers for a well‐powered analysis. Additionally, we did not perform separate analyses based on therapy intensity (intensive vs. low‐intensity), although >70% of patients received low‐intensity treatments, allowing for a degree of generalizability. To preserve the integrity of the observed data, we deliberately chose not to impute missing values.

In conclusion, the majority of *TP53* mutated myeloid neoplasms with ≥5% blasts exhibit a −5/5q as well as a complex karyotype. The overall cytogenetic complexity in context of complex karyotype confounds −5/5q impact. Our study underscores the value of karyotype in high‐risk cohort of TP53 mutated myeloid neoplasm as del(5q) is associated with a negative prognostic impact, particularly in cases with less than 20% blasts and those with *TP53* ^*SH*^ allelic state.

## AUTHOR CONTRIBUTIONS

**Irfan Yasin:** Resources; Writing—original draft; Writing—review & editing; Data curation; and Supervision. **Anna Stengel:** Resources; Data curation; Writing—review & editing; Formal analysis; and Writing—original draft. **Haipeng Shao:** Data curation; Writing—review & editing; and Resources. **Amandeep Kaur:** Data curation; Resources; and Writing—review & editing. **Emily F. Mason:** Data curation; Writing—review & editing; and Resources. **Pranav P. Patwardhan:** Data curation; Writing—original draft; and Resources. **Nathanael G. Bailey:** Data curation; Writing—review & editing; and Resources. **Sharmila Ghosh:** Data curation; Writing—review & editing; and Resources. **Kedar V. Inamdar:** Data curation; Writing—review & editing; and Resources. **Anand A. Patel:** Data curation; Writing—review & editing; and Resources. **Madhavi Pandiri:** Data curation; Writing—original draft; Writing—review & editing; and Resources. **Jingjing Zhang:** Data curation; Writing—review & editing; and Resources. **Payal Sojitra:** Data curation; Writing—review & editing; and Resources. **Hamza Tariq:** Data curation; Writing—review & editing; and Resources. **Zenggang Pan:** Writing—review & editing; Data curation; and Resources. **Danica Wiredja:** Data curation; Resources; and Writing—review & editing. **Peng Wang:** Conceptualization; Resources; Formal analysis; and Writing—review & editing. **Melissa Y. Tjota:** Resources; Formal analysis; Writing—review & editing; and Data curation. **Jeremy P. Segal:** Resources; Data curation; and Writing—review & editing. **Hong Chang:** Resources; Data curation; and Writing—review & editing. **David A. Sallman:** Data curation; Resources; and Writing—review & editing. **Daniel A. Arber:** Resources and Writing—review & editing. **Ayalew Tefferi:** Resources; Writing—review & editing; and Data curation. **Talha Badar:** Resources; Formal analysis; Writing—original draft; and Writing—review & editing. **Anamarija M. Perry:** Resources; Writing—original draft; and Writing—review & editing. **Claudia Haferlach:** Data curation; Resources; and Writing—review & editing. **Angela M. Lager:** Resources; Formal analysis; Investigation; and Writing—review & editing. **Girish Venkataraman:** Conceptualization; Methodology; Software; Data curation; Investigation; Supervision; Visualization; Project administration; Resources; Writing—original draft; Writing—review & editing; and Formal analysis. 

## CONFLICT OF INTEREST STATEMENT

A. Patel, Honoraria from Sobi, AbbVie, BMS| Research support from Pfizer, Sumitomo, Kronos Bio; D. Sallman, Research funding from Aprea Therapeutics and Jazz Pharmaceuticals; Consulting and/or other fees from Abbvie, Agios, Akesobio Avencell Europe GmbH, Bluebird Bio, BMS, Curis, Gilead Sciences, Incyte, Intellia Therapeutics, Janssen Global Services, Jazz Pharmaceuticals, Kite Pharma, Molecular Partners AG, Novartis, Servier Pharmaceuticals, Shattuck Labs, Syndax Pharmaceuticals, Syros Pharmaceuticals, and Takeda Pharmaceutical. MS reports serving on advisory boards for Novartis, Kymera, Sierra Oncology, GSK, Rigel, BMS, Sobi, and Syndax; C. Haferlach, Part ownership of MLL Münchner Leukämielabor.; I. Yasin, A. Stengel, H. Shao, A. Kaur, E. Mason, P. Patwardhan, N. Bailey, S. Ghosh, K. Inamdar, M. Pandiri, J. Zhang, P. Sojitra, H. Tariq, Z. Pan, D. Wiredja, P. Wang, M. Tjota, J. Segal, H. Chang, D. Arber, A. Tefferi, T. Badar, A. Perry, A. Lager, G. Venkataraman have no disclosure(s).

## DATA SHARING STATEMENT

Please address data requests to the corresponding author via email: girish.venkataraman@uchospitals.edu

## Data Availability

The data that support the findings of this study are available from the corresponding author upon reasonable request.
